# Fatal Hypogammaglobulinemia 3 Years after Rituximab in a Patient with Immune Thrombocytopenia: An Underlying Genetic Predisposition?

**DOI:** 10.1155/2019/2543038

**Published:** 2019-12-28

**Authors:** Jean-François Viallard, Marie Parrens, Frédéric Rieux-Laucat

**Affiliations:** ^1^Internal Medicine Department, Hôpital Haut-Lévêque, Bordeaux University Hospital, Avenue de Magellan, Pessac, France; ^2^Pathology Department, Hôpital Haut-Lévêque, Bordeaux University Hospital, Avenue de Magellan, Pessac, France; ^3^INSERM UMR 1163, Laboratory of Immunogenetics of Pediatric Autoimmune Diseases, Paris Descartes-Sorbonne, Paris Cité University, Imagine Institute, Paris, France

## Abstract

We report the case of a young woman who developed, 3 years after stopping Rituximab (RTX) prescribed for immune thrombocytopenia (ITP), a severe immunodeficiency leading to fatal pulmonary Epstein–Barr virus-positive diffuse large B-cell lymphoma. Genetic analysis led us to identify four missense mutations known to affect immune-deficiency–associated genes (FAS-ligand (*FASL*) gene (p.G167R); perforin-1 (*PRF1* (p.R55C) gene; the Bloom syndrome RecQ-Like helicase (*BLM*) gene and the Moesin (*MSN*) (p.A122T) gene). The heterozygous mutation in the *FASL* gene, not present in the Genome Aggregation Database or ClinVar database, could suggest atypical Autoimmune LymphoProliferative Syndrome and its role in this patient's immunodepression is discussed. This observation strengthens the role of *FASL* gene mutation in severe clinical phenotypes of primary immune deficiency and raises new questions about the genetic background of ITP occurring in young people in a context of immunodeficiency.

## 1. Introduction

Immune thrombocytopenic purpura (ITP) is a frequent complication in patients with Autoimmune LymphoProliferative Syndrome (ALPS), occurring in up to 40% of patients [1, 2]. ALPS, caused by mutations in the *FAS* apoptotic pathway [1], can be diagnosed in young adults, often at the onset of autoimmune cytopenia, while chronic lymphoproliferation is less prominent than in childhood [3]. In a recent study, Vandrovcova et al. screened a cohort of 130 adult patients with persistent or chronic primary ITP for mutations in the *FAS* gene and identified two potentially functional mutations in two patients with atypical ALPS clinical features [4]. Otherwise, Evans syndrome, characterized by the combination of autoimmune hemolytic anemia and ITP, is potentially genetically determined in at least 65% of cases in pediatric population [5]. So, these observations raise the possibility of a genetic defect in ITP young people, mostly in a context of immunodeficiency, and ALPS mutations must primarily be investigated. Then, we report another original case of a young woman who developed an immunodeficiency leading to fatal pulmonary Epstein–Barr virus (EBV)-positive diffuse large B-cell lymphoma (DLBCL), 3 years after stopping Rituximab (RTX) prescribed for ITP. Genetic testing led us to identify an unknown heterozygous mutation in the FAS(CD95)-ligand gene *(FASLG)* and its role in this patient's immunodepression is discussed.

## 2. Clinical Case

In July 2015, a 24-year-old woman was referred to our Department of Internal Medicine for a high fever (39°C) lasting 3 days, fatigue, myalgias, chills, and vomiting. She had been followed since 2008 for primary ITP, initially treated with oral prednisone (1 mg/kg/day), which achieved complete remission. Because of occasional severe relapses (two between 2009 and 2012, with gynecological bleeding), she was subsequently treated with Intravenous Immunoglobulin (IVIg) with good responses. In January 2012, at age 21, she suffered a severe relapse, again justifying the use of IVIg and corticosteroids. At that time, she had detectable autoimmunity with an antinuclear antibody titer of 1 : 250 (anti-SSA specificity but without any sign suggestive of lupus) and platelet-directed anti-glycoprotein IIb/IIIa antibodies. In June 2012 (baseline), a new IVIg cycle was administered, followed by RTX (375 mg/m^2^ once-a-week for 4 consecutive weeks). A complete platelet response was obtained within 6 weeks and, at the last follow-up (March 2015), her blood platelet level was normal (321 × 10^9^/L) without treatment. Before RTX infusion (June 2012), her blood total gamma-globulin level >3 months before IVIg infusion had been normal (8.9 g/L) but she was lymphopenic (total lymphocytes: 0.513 × 10^9^/L), while her peripheral blood lymphocyte count had been normal at ITP diagnosis (1.199 × 10^9^/L). The previously available phenotype profiles of her peripheral circulating lymphocytes are reported in Table 1. No infection occurred during the 3 years following the last RTX administration and she remained clinically well at biannual consultations in our department.

At admission, in July 2015, at age 24, her temperature was 39.2°C and she complained of lower abdominal pain, vomiting but without diarrhea; her physical examination was normal. Laboratory tests showed elevated C-reactive protein (CRP: 114 mg/L, normal range (NR): <5 mg/L), hepatic cytolysis (aspartate aminotransferase: 144 U/L, NR: 7–40 U/L; alanine aminotransferase: 265 U/L, NR: 5–50 U/L) and cholestasis (alkaline phosphatase: 389 IU/L, NR: 40–130 U/L; *γ*-glutamyltranspeptidase: 698 U/L, NR: 5–38 U/L). The hemogram revealed agranulocytosis and thrombocytopenia (leukocytes: 4.9 × 10^9^/L; neutrophils: 0.02 × 10^9^/L; hemoglobin: 14.5 g/dL; platelets: 46 × 10^9^/L). Bone-marrow aspirate showed normal density, several megakaryocytes but hypoplastic granulopoiesis in blocked maturation, without blasts. Computed-tomography (CT) scans of the thorax and abdomen were normal. Once urine and blood samples had been collected, empirical ceftazidime and gentamicin were begun, and achieved apyrexia within 48 h. After 46 h, blood cultures came back positive for *Campylobacter jejuni*, and the identical strain isolated from the patient's feces; amoxicillin–clavulanic acid (1 g 3 times a day for 2 weeks) was prescribed. Seven days postadmission, CRP, neutrophil and platelet levels returned to normal (3 mg/L, 6.61 × 10^9^/L and 521 × 10^9^/L, respectively); blood cultures were negative; and liver function was improved.

Laboratory evaluation for immunodeficiency yielded: negative serology for human immunodeficiency viruses-1 and -2, very low serum Ig levels (Table 1) and no circulating B cells. Flow-cytometry quantification of T-cell subsets found expansion of circulating CD4^+^ T lymphocytes expressing surface DR, suggesting their activation; low natural killer cells and CD8^+^ T-lymphocyte levels. Urine immunofixation electrophoresis was negative. Ig-replacement therapy was started but not pursued by the patient.

Ten months later (May 2016, age 25) she was hospitalized for a 15-day low-grade fever, weight loss, nonproductive cough and progressive dyspnea. CRP, lactate dehydrogenase and *β*_2_-microglobulin levels were 40 mg/L, 340 U/L (NR 5–240 U/L) and 3.53 mg/L (NR 1.2–2.5 mg/L), respectively. As shown in Table 1, hypogammaglobulinemia, CD19^+^ B lymphopenia and low peripheral CD8^+^ T-cell count persisted but without T activation, as assessed by HLADR-positivity. CT scans visualized bilateral diffuse infiltrates, ground-glass opacities and large nodules. Bronchoalveolar lavage analysis revealed *Pneumocystis jiroveci* cysts with positive polymerase chain reaction (PCR) (4,000 copies/mL); high-dose trimethoprim–sulfamethoxazole and corticosteroids were prescribed. Searches for other pathogens, including *Mycobacterium tuberculosis*, atypical mycobacteria, cytomegalovirus, *Cryptococcus* and *Aspergillus* species, were negative. At that time, her bone-marrow biopsy was normal.

**Figure 1:**
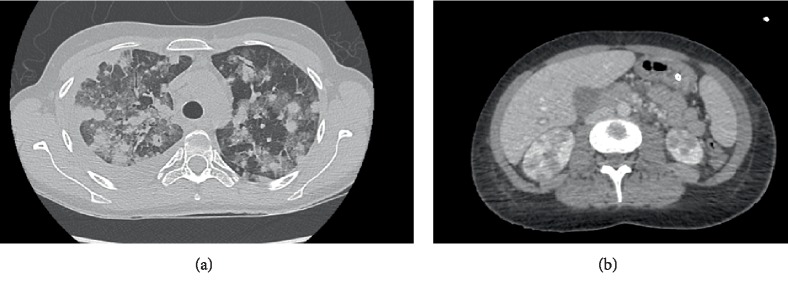
Imaging. (a) Pulmonary computed-tomography (CT) scan showing diffuse bilateral infiltrates. (b) CT with intravenous contrast showing enlargement of both kidneys with bilateral renal nodules.

**Figure 2:**
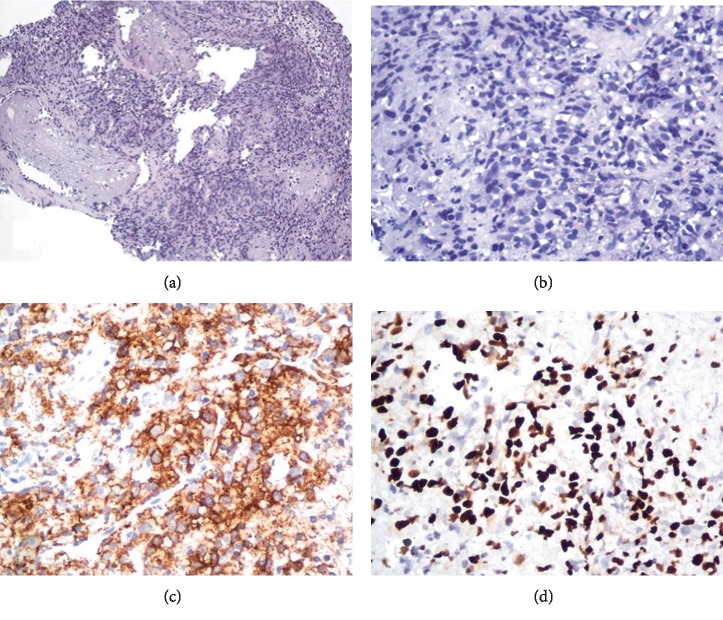
Histology. (a) Lymphomatous infiltration in the bronchial mucosa (hematoxylin–eosin (HE) staining, ×40). (b) Large diffuse large B-cell lymphoma (DLBCL) cells characterized by nuclei with prominent nucleoli on a background of tumoral necrosis (HE, ×400). (c) CD20+ labeling of the DLBCL cells. (d) Epstein–Barr virus-encoded RNA-1 expressed by DLBCL cells.

Despite appropriate antibiotics and clinical improvement, thoracic CT scans revealed worsened dense infiltrates (Figure 1(a)), pleural effusions, hepatosplenomegaly and nodular lesions of both kidneys (Figure 1(b)). A new bronchoscopy with biopsies found CD20+ large lymphomatous cell infiltration (Figure 2) in bronchi. Those large atypical lymphoid tumor cells were CD10^−^BCL-6^−^ and MUM1^+^BCL-2^+^, with an 80% Ki-67–proliferation index on immunolabeling. EBV, as assessed by in situ hybridization with an EBV-encoded small RNA probe, was diffusely positive in about 80% of tumor cells (Figure 2). The FISH assay for *MYC* gene rearrangement (MYC FISH DNA Probe, Split Signal, (Y5410), Dako, Locus 8q24) was negative. EBV-positive DLBCL with a nongerminal center phenotype was diagnosed without bone-marrow infiltration. Circulating EBV-DNA was positive (2,430,000 IU/mL). DLBCL treatment consisted of RTX, cyclophosphamide, doxorubicin, vincristine and prednisone. Even with EB viremia becoming negative, she developed fever, cytopenias, liver damage and neurological manifestations, as a consequence of her prominent bone-marrow hemophagocytosis. Unfortunately, she died of multiorgan failure at age 25.

## 3. Genetic Analyses

DNA extracted using standard methods [6] from the bone-marrow sample obtained when she had *Pneumocystis jiroveci* pneumonia was subjected to next-generation sequencing. Exons from genomic DNA underwent PCR amplification according to standard protocols [5]. The PCR amplificons were then sequenced in both directions and analyzed with ApE-A plasmid Editor v2.0.45.

Using 1–3 *µ*g of DNA according to the manufacturer's protocol (Ovation Ultralow, Nugen Technologies), we constructed Illumina-compatible, pre-capture, bar-coded, genomic DNA libraries. Equimolar concentrations of several such pre-capture libraries were pooled and used with biotinylated probes from the SureSelect panel (Agilent, Les Ulis, France) for the capture process, and, finally, sequenced on an Illumina HiSeq2500 (paired-end sequencing 130 × 130 bases, high-throughput mode). Using the Burrows–Wheeler alignment version 0.6.2.13, the read sequences were aligned to reference human genome hg19. For each sample, depth of coverage reached at least a mean 300x; it covered ≥97% of the panel regions at 15× and ≥90% of them at least at 30x.

To identify all high-throughput sequencing-generated single nucleotide polymorphisms and insertions/deletions, the Genome-Analysis Toolkit and an in-house program (Polyweb) were used to filter the variants. We excluded variants representing >1% of the populations previously reported in public databases: e.g., the 1000-genomes project, *Exome Variant Server*, double-base single nucleotide polymorphisms and in-house–identified variants (4047 exomes). The exomes were mined for variations, i.e., substitutions and insertions/deletions in the coding regions, splice sites and essential splice sites in the flanking introns. Coding-region silent mutations were excluded from this analysis.

The search for variants known to be associated with primary immune deficiency (PID) failed to reveal rare recessive or heterozygous mutations (i.e., in cytotoxic T-lymphocyte antigen-4 (*CTLA4*), phosphatidylinositol-4,5-bisphosphate 3-kinase, catalytic subunit-*δ* (*PIK3CD*), phosphatidylinositol-4,5-bisphosphate 3-kinase, receptor-1 (*PIK3R1*), X-linked inhibitor of apoptosis (*XIAP*) and *FAS*). Our analysis focused on rare private variants with generally deleterious predictive scores: SIFT and PolyPhen2, the two most commonly used algorithms to predict negative impact on protein structure, and the Combined Annotation-Dependent Depletion (CADD) score >20. Together they identified 69 unique private variants, four of which were missense mutations known to affect immune-deficiency–associated genes (Table 2).

The FAS-ligand (*FASL*) gene (p.G167R), the first missense gene to be identified, was not in the Genome Aggregation Database (gnomAD) or ClinVar database. It is predicted to interfere with *FASL* extracellular domains. The *PRF1* (p.R55C) gene, which codes for perforin-1, was the second missense mutation. Among its 58 entries in the gnomAD, one was homozygous. Familial hemophagocytotic lymphohistiocytosis is associated with homozygous *PRF1* mutations. Heterozygous (58 times) and homozygous (9 times) variants of the Bloom syndrome RecQ-Like helicase (*BLM*) gene, the third missense mutation, were found in the gnomAD. Its homozygous mutations are linked to the Bloom syndrome. In light of the frequency of the last two mutations in the public gnomAD, especially homozygous mutants, we considered them to be polymorphic variants. The fourth missense variant affects the membrane-organizing extension spike protein, which encodes Moesin (*MSN*) (p.A122T); it has already been listed three times in the gnomAD. *MSN* is located on the X chromosome. Although no one carrying homozygous *MSN* has yet been reported, hemizygous *MSN* mutations have been reported in patients with X-linked PID [7]. The deleterious prediction algorithms used for this variant yielded totally discordant findings: SIFT indicated it is innocuous, PolyPhen2 suggested it is probably.

## 4. Discussion

This case is uncommon because of the prolonged hypogammaglobulinemia persisting after RTX discontinuation and then, secondarily, after *Campylobacter* infection, the appearance of uncontrolled EBV-induced lymphoproliferation. Several papers alerted clinicians to the risk of symptomatic hypogammaglobulinemia >2 years after RTX administration to ITP patients with initially normal Ig levels (reviewed in [8]). So, did our patient have transient RTX-induced hypogammaglobulinemia or RTX-unmasked underlying humoral PID? The latter hypothesis seems the most likely. Indeed, B-cell lymphopenia is certainly observed for several months after RTX administration but usually not for >3 years [9]. Shortly before the first RTX infusion, our patient was already B-cell lymphopenic. It would have been highly informative to have a detailed naïve/memory B-cell phenotype, notably the percentage of switched-memory B cells, as it is usually low in Common Variable Immunodeficiency (CVID) with autoimmune manifestations. Many primary humoral defects, e.g. CVID, can be preceded and/or revealed by autoimmune manifestations like ITP, as for our patient. Lastly, the authors reporting extremely severe hypogammaglobulinemia 7 years after treatment of indolent lymphoma [10] postulated worsening of preexisting CVID.

The discovery of our patient's *FASL* gene mutation could suggest atypical ALPS. Indeed, ALPS has been associated with recessive and dominant *FASL* gene mutations [11–14]. It is predicted to interfere with *FASL* extracellular domains. However, the causality of those heterozygous *FASL* gene mutations was not definitively confirmed [15]. The variant c.499G>A present in our patient has never been described in general population nor in the context of autoimmune disease, and may be a “probably pathogenic” mutation. Some ALPS *FAS*-mutated patients experience their first symptoms, particularly autoimmune cytopenia, in adulthood [3]. Unfortunately, plasma biomarkers, now routinely assayed during the prediagnosis work-up for ALPS-FAS, such as soluble FASL (sFASL) or interleukins 10 and 18 [16], can no longer be determined for our deceased patient. It is therefore difficult to consider an ALPS diagnosis for our patient because she had neither splenomegaly nor lymphadenopathy, and no accumulation of a peripheral population of TCR*αβ*^+^CD4^−^CD8^−^ T cells (referred to as double-negative (DN) T cells) (Table 1), the pathognomonic feature of ALPS, was observed. Moreover, her blood vitamin-B_12_ level was normal. However, the two ITP patients described by Vandrovcova et al. with nonsynonymous heterozygous variants in FAS had few classical features of ALPS [4]. For example, the patient with the p.Cys199Arg mutation (located outside the death domain and resulting in aberrant signaling), had no hepatosplenomegaly nor lymphadenopathy and slightly increased *αβ*-DN T cells [4]. These observations raise possible relation between unusual ALPS phenotypes and novel types of mutations in both FAS and the death ligand *FasL*. Although putative contributions of the *FASL* and *PRF1* genes mutations could be advanced for our patient, we have no possible logical pathophysiological mechanisms to explain her fatal disease.

A potential role of the *MSN* mutation could have been contributory, given the immune deficiency observed in hemizygous patients [7]. Heterozygous females with X-linked gene mutations have developed attenuated or even severe clinical [17, 18], inhibitor of nuclear factor (NF) *κ*B kinase subunit-*γ*/NF-*κ*B essential modulator (*IKBKG/NEMO*) gene mutations, with incontinentia pigmenti being the most well-known example. While deleterious mutations are usually lethal in males, the Moesin-associated disease develops variably in heterozygous females. Moreover, *IKBKG* hypomorphic hemizygous mutations are associated with ectodermal dysplasia, anhidrotic lymphedema and immunodeficiency. More recently, *XIAP* mutations, associated with an EBV-triggered lymphoproliferative syndrome in hemizygous males [19], have been found in heterozygous females with erythroderma nodosa and/or inflammatory bowel disease resulting from random X-inactivation [20]. Although such epigenetic events have never been reported in the few known cases of *MSN*-mutation–associated immunodeficiency, that hypothesis cannot be excluded at present. Further analyses of blood samples would have been needed to support/refute it.

In conclusion, our observation strengthens the involvement of *FASL* gene mutation as causative agent of severe clinical phenotypes of PID, and raises new questions about the genetic background of ITP occurring in young people in a context of immunodeficiency. Wide-ranging genetic screening should be then proposed to these patients, focusing on mutations known to be strong risk factors for autoimmune cytopenia such as FAS and FASL mutations. All patients with autoimmune cytopenia should have their blood Ig levels determined, because ITP can be the first manifestation of PID, and monitored after a disease-modifying treatment, like RTX or splenectomy, is undertaken. Henceforth, early PID diagnosis in young patients is challenging for critically ill patients to choose the best adapted regimen, including targeted-immunotherapy or even hemopoietic stem-cell transplantation.

## Figures and Tables

**Table 1:** Summary of the patient's immunological profile.

Parameter	Normal range	At ITP diagnosis (2008)	6 months before RTX	Baseline^∗^	6 months post-RTX	Jul 2015	Nov 2015	May 2016
Blood hemoglobin, g/dL	12–16	14.8	12.1	13.7	14.3	14.5	13.6	11.9
Blood platelets, G/L	150–400	3	2	24	368	49	322	404
Blood leukocytes, /mm^3^	4000–10,000	6,300	3,750	5,800	4,890	4,100	5,200	6,600
Blood neutrophils, /mm^3^	2,500–7,500	4,570	2,670	4,950	3,590	120	3,100	5,430
Monocytes, /mm^3^	200–1,000	370		190	270	600	370	400
Total blood gamma-globulins, g/L	7.8–16	8.4		8.9	ND	2.3	2.6	2.8
IgG	6.90–14	ND	ND	ND	ND	2.19	2.37	2.86
IgA	0.70–3.70	ND	ND	ND	ND	0.477	0.52	0.51
IgM	0.40–2.40	ND	ND	ND	ND	0.11	0	0.2
Total lymphocytes/mm^3^		1,199	513	589	705	3,000	861	402
CD19^+^ B cells, %	11–16	18.13	ND	16.75	0	0	0	0
CD19^+^ B cells/mm^3^	200–400	217	ND	99	0	0	0	0
CD3^+^ T cells/mm^3^	1,100–1,700	923	422	453	607	2,949	757	317
CD4^ +^T cells/mm^3^	700–1,100	745	330	359	456	2,774	589	252
CD8^+^ T cells/mm^3^	500–900	138	69	74	131	132	151	54
CD4^+^/CD8^+^ ratio		5.39	4.78	4.85	3.5	21	12	4.6
CD3^+^CD4^–^CD8^–^T cells/mm^3^		37	21	18	17	43	40	7
*γδ* T Lymphocytes/mm^3^		ND	ND	ND	6	ND	ND	3
CD4^+^DR^+^ T cells, %		4.03	7.88	4.5	3.7	59.1	ND	9.59
CD8^+^DR^+^ T cells, %		16.67	29	17.8	5.5	19.7	ND	12.96
CD3^–^CD16^+^CD56^+^ NK/mm^3^	200–400	ND	ND	18	7	27	ND	20

^∗^Baseline, 2012 just prior to first rituximab infusion. ND: not determined. NR: normal range, NK: natural killers.

**Table 2:** Sequencing results of heterozygous PID-related variants affecting the *FASL*, *PRF1*, *BLM*, and *MSN* genes.

Gene	Consequence	Transcript	Exon	cDNA_Pos	Cds_Pos	AA	Protein_Pos	Nomenclature	PolyPhen	SIFT	CADD
*FASL*	Missense	ENST00000367721_1	4	683	499	G/R	167	c.499G>A	Probably damaging (score: 1)	Deleterious (score: 0)	27.5
*PRF1*	Missense	ENST00000441259_10	2	324	163	R/C	55	c.163C>T	Probably damaging (score: 0.959)	Deleterious (score: 0)	26.6
*BLM*	Missense	ENST00000355112_15	2	161	43	R/C	15	c.43C>T	Probably damaging (score: 0.939)	Deleterious (score: 0.02)	34
*MSN*	Missense	ENST00000360270_X	4	536	364	A/T	122	c.364G>A	Possibly damaging (score: 0.718)	Benign (score: 0.09)	25.6

## References

[1] Rieux-Laucat F. (2015). What’s up in the ALPS. *Current Opinion in Immunology*.

[2] Rao V. K. (2015). Approaches to managing autoimmune cytopenias in novel immunological disorders with genetic underpinnings Like autoimmune lymphoproliferative syndrome. *Frontiers in Pediatrics*.

[3] Lambotte O., Neven B., Galicier L. (2013). Diagnosis of autoimmune lymphoproliferative syndrome caused by FAS deficiency in adults. *Haematologica*.

[4] Vandrovcova J., Salzer U., Grimbacher B. (2019). FAS mutations are an uncommon cause of immune thrombocytopenia in children and adults without additional features of immunodeficiency. *British Journal of Haematology*.

[5] Hadjadj J., Aladjidi N., Fernandes H. (2019). Pediatric Evans syndrome is associated with a high frequency of potentially damaging variants in immune genes. *Blood*.

[6] Miller S. A., Dykes D. D., Polesky H. F. (1988). A simple salting out procedure for extracting DNA from human nucleated cells. *Nucleic Acids Research*.

[7] Lagresle-Peyrou C., Luce S., Ouchani F. (2016). X-linked primary immunodeficiency associated with hemizygous mutations in the moesin (*MSN*) gene. *Journal of Allergy and Clinical Immunology*.

[8] Levy R., Mahévas M., Galicier L. (2014). Profound symptomatic hypogammaglobulinemia: a rare late complication after rituximab treatment for immune thrombocytopenia. Report of 3 cases and systematic review of the literature. *Autoimmunity Reviews*.

[9] Casulo C., Maragulia J., Zelenetz A. D. (2013). Incidence of hypogammaglobulinemia in patients receiving rituximab and the use of intravenous immunoglobulin for recurrent infections. *Clinical Lymphoma Myeloma and Leukemia*.

[10] Walker A. R., Kleiner A., Rich L. (2008). Profound hypogammaglobulinemia 7 years after treatment for indolent lymphoma. *Cancer Investigation*.

[11] Del-Rey M., Ruiz-Contreras J., Bosque A. (2006). A homozygous Fas ligand gene mutation in a patient causes a new type of autoimmune lymphoproliferative syndrome. *Blood*.

[12] Magerus-Chatinet A., Stolzenberg M. C., Lanzarotti N. (2013). Autoimmune lymphoproliferative syndrome caused by a homozygous null FAS ligand (*FASLG*) mutation. *Journal of Allergy and Clinical Immunology*.

[13] Nabhani S., Hönscheid A., Oommen P. T. (2014). A novel homozygous Fas ligand mutation leads to early protein truncation, abrogation of death receptor and reverse signaling and a severe form of the autoimmune lymphoproliferative syndrome. *Clinical Immunology*.

[14] Bi L. L., Pan G., Atkinson T. P. (2007). Dominant inhibition of Fas ligand-mediated apoptosis due to a heterozygous mutation associated with autoimmune lymphoproliferative syndrome (ALPS) Type Ib. *BMC Medical Genetics*.

[15] Pauly E., Fritzsching B., Dechant M., Fellenberg J., Scheuerpflug C. G., Debatin K.-M. (2006). Analysis of the CD95 ligand gene in 20 children with autoimmune lymphoproliferative syndrome (ALPS). *Blood*.

[16] Oliveira J. B., Bleesing J. J., Dianzani U. (2010). Revised diagnostic criteria and classification for the autoimmune lymphoproliferative syndrome (ALPS): report from the 2009 NIH International Workshop. *Blood*.

[17] Döffinger R., Smahi A., Bessia C. (2001). X-linked anhidrotic ectodermal dysplasia with immunodeficiency is caused by impaired NF-κB signaling. *Nature Genetics*.

[18] Zonana J., Elder M. E., Schneider L. C. (2000). A novel X-linked disorder of immune deficiency and hypohidrotic ectodermal dysplasia is allelic to incontinentia pigmenti and due to mutations in *IKK-gamma* (NEMO). *American Journal of Human Genetics*.

[19] Rigaud S., Fondanèche M. C., Lambert N. (2006). XIAP deficiency in humans causes an X-linked lymphoproliferative syndrome. *Nature*.

[20] Dziadzio M., Ammann S., Canning C. (2015). Symptomatic males and female carriers in a large Caucasian kindred with XIAP deficiency. *Journal of Clinical Immunology*.

